# Prediction of fat-free mass from body surface area in young basketball players

**DOI:** 10.1186/s13102-024-00857-x

**Published:** 2024-03-06

**Authors:** Anderson Marques de Moraes, Ruben Vidal-Espinoza, Raiany Rosa Bergamo, Rossana Gómez-Campos, Evandro de Lazari, Luis Felipe Castelli Correia de Campos, Jose Sulla-Torres, Marco Cossio-Bolaños

**Affiliations:** 1 Department of Physical Education, School of Sports, Pontifical Catholic University of Campinas, Campinas, Brasil; 2 Universidad Católica Silva Henriquez, Santiago, Chile; 3 Laboratory of Growth and Development (LabCred), Pediatrics Research Center (CIPED), Sao Paulo, Brazil; 4 Faculty of Medical Sciences (FCM), State University of Campinas (Unicamp), Campinas, Sao Paulo, Brazil; 5 Universidad San Ignacio de Loyola, Lima, Perú; 6 Faculdade de Educação Física - UNICAMP, Campinas, Brasil; 7 Universidad del Bio Bio, Chillán, Chile; 8 Núcleo de Investigación en Ciencias de la Motricidad Humana, Universidad Adventista de Chile, Chillán, Chile; 9 Universidad Católica de Santa María, Arequipa, Peru

**Keywords:** Fat free mass, Equations, Body surface area, Basketball players, Youth

## Abstract

**Background:**

Fat Free Mass (FFM) is an important and essential indicator in various sports populations, since greater muscle and bone mass generates greater strength, endurance and speed in athletes.

**Objective:**

The purpose of the study was to validate Body Surface Area (BSA) as an anthropometric indicator to estimate FFM in young basketball players.

**Methods:**

A descriptive cross-sectional study was carried out in 105 male basketball players of the Brazilian Basketball Confederation of Sao Paulo (Campinas), Brazil. The age range was 11 to 15 years. Weight and height were evaluated. BSA, body mass index (BMI) and maturity status (MS) were calculated. Total body scanning was performed by dual X-ray absorptiometry (DXA). The components were extracted: Fat mass (FM), Fat free mass (FFM), percentage of fat mass (%FM) and bone mass (BM). The data were analyzed using the correlation coefficient of concordance (CCC) in terms of precision and accuracy.

**Results:**

Three regression equations were generated: equation 1 had age and body weight as predictors [FFM= -30.059+(2.926*age)+(0.625*Weight)] (R^2^ = 92%, precision = 0.96 and accuracy = 0.99), equation 2 used age and BSA [FFM=-45.719+(1.934*age)+(39.388*BSA)] (R^2^ = 94%, precision = 0.97 and accuracy = 0.99) and equation 3 was based on APHV and BSA [FFM=-15.284+(1.765*APHV)+(37.610*(BSA)] (R^2^ = 94%, precision = 0.96 and accuracy = 0.99).

**Conclusions:**

The results suggest the use of anthropometric equation using decimal age and BSA to estimate FFM in young basketball players. This new method developed can be used to design, evaluate and control training programs and monitor the weight status of athletes.

## Background

Fat-free mass (FFM) refers to the amount of non-fat body tissue, which includes muscle, bone, water and organs. It is used as an indicator of nutritional and health status in various populations [1]. It is characterized as an important indicator of body composition and health in populations of different ages and physical activity levels [2]. It is generally used to assess changes in body composition during weight loss and physical training, and has been related to various health outcomes, such as mortality, cardiovascular disease and diabetes [3]. Its assessment is often performed from direct (cadaveric analysis), indirect (chemical and physical) and doubly indirect (anthropometry and bio-impedance) methods [4, 5].

In fact, regardless of the method used, its use and application are considered essential in sports populations. Because greater muscle and bone mass generates greater strength, endurance and speed in athletes [6] and, consequently, greater physical performance. In the specific case of basketball, some studies [7–9], have shown that components of body composition are directly related to multiple variables of motor performance, pointing out the positive contribution of fat-free mass. The existence of a relationship has been established, where success in sports performance depends on the player’s jumping ability simultaneously with shooting or rebounding [10]. The vertical jumping performance of a player has a direct impact on his level of play, as the former is an integral part of different game movements such as rebounding, shooting at the basket, shooting at the basket, dunking and rebounding [11].

From that perspective, several studies in recent years have highlighted that anthropometric measures such as bone breadths (such as femur or humerus), body girths (arm, leg girths or thigh girths), and skinfolds (triceps and subscapular), are classic indicators that serve to estimate FFM in non-sporting populations of children, youth and adults, respectively [12–14].

However, to our knowledge, there is a wide variety of studies that have reported equations estimating FFM in young athletes in various sport modalities and mainly in soccer [15–18].

To date, no equations based on anthropometric measures that predict FFM in young basketball players have been identified. Thus, there is currently an urgent need to refine anthropometric methods that provide information beyond diameters, circumferences and skinfolds.

In this context, it is necessary to develop FFM prediction equations that are less time-consuming and fast in their measurements and calculations. In recent years, some studies have suggested that Body Surface Area (BSA) could be an appropriate indicator to estimate FFM in children and adolescent athletes [19] and non-athletes [20], since there is a positive relationship between both variables.

In fact, BSA has been classically defined as a measure of the extent of an individual’s skin surface área [21] and represents human dimensionality and predicts metabolic activity in clinical applications and metabolic heat production in physiology [22]. It is calculated from the height and weight of an individual, using classical mathematical formulas, such as the Dubois formula [23] or others. These equations have previously been allometrically adjusted where the geometric calculations applied normalize weight and height according to body size. The Dubois formula was used since previous studies highlight its use in athletes especially in soccer players, basketball players and volleyball players [24, 25].

Consequently, this study presupposes that BSA could be an excellent predictor of FFM in young asketball players. Since, the proposal of new predictive equations based on non-invasive methods could be a very useful alternative for clubs and professionals working in the detection and selection of sports talent. Therefore, the objective of the study was to validate the BSA as an anthropometric indicator to estimate the FFM of young Brazilian basketball players.

## Materials and methods

### Subjects

A descriptive cross-sectional study was carried out on 105 male basketball players of the Brazilian Basketball Confederation of Sao Paulo (Campinas), Brazil. The sample selection was non-probalistic (accidental), the youngsters belonged to 10 representative clubs, whose age range was from 11 to 15 years old.

The young people participated in the study on a voluntary basis and had 3 years of experience in the sport. Those who were within the established age range and those who were duly registered with the Brazilian basketball confederation were included in the study. Young people with any type of sports injury that prevented anthropometric evaluations and dual X-ray absorptiometry (DXA) scanning were excluded from the study. Also, children categorized as pre-pubertal (according to maturity status) were excluded of study. All parents and/or guardians of the youngsters signed the informed consent. The youth also signed the informed consent form. The study was approved by the Research Ethics Committee of the State University of Campinas UNICAMP (CAAE: 79718417.0.0000.5404). All evaluation procedures were performed in accordance with the Helsinki declaration for human subjects.

### Techniques and procedures

Anthropometric measurements and DXA scanning were performed at the hospital of the faculty of medical sciences of the university. All measurements were performed in the morning period from Monday to Friday.

For the evaluation process, a team of 4 evaluators with extensive experience in anthropometric evaluations and with ISAK certification (02 evaluators) and DXA scanning (02 evaluators) was formed.

The concordance between the evaluators of anthropometric variables and DXA scanning was verified through the Kappa Coefficient. The kappa coefficient values obtained reflected an almost perfect concordance strength [26]. It was 0.93 for the anthropometrist and 0.94 for the DXA evaluators, confirming a high degree of agreement between them.

The anthropometric measurements of weight and height were evaluated without shoes and with as little clothing as possible, following the recommendations of Ross, Marfell-Jones [27]. Body weight (kg) was measured using an electronic scale (Tanita, United Kingdom) with a scale from 0 to 150 kg and with 100 g accuracy. Standing height with a portable stadiometer (Seca Gmbh & Co. KG, Hamburg, Germany) accurate to 0.1 mm, according to the Frankfurt plan.

The FFM analysis was performed using the standard method by iDXA. The equipment used was: (GE Healthcare Lunar, Madison, WI, USA) and enCore™2011 software version 13.6 (GE Healthcare Lunar). Total body measurements, including the head, were performed.

Prior to the scan, the athletes were prohibited from wearing jewelry and the presence of some types of metal on the body, which should be removed before the scan. To start the scan, the athletes were instructed to remain in the supine position with arms extended at the sides of the body and with the knees and ankles fastened with a Velcro strap (to ensure the predetermined position). One of the evaluators aligned the reference points according to the lines shown by the software. Body composition indicators [Fat free mass (FFM), Fat mass (FM), percentage of fat mass (%FM) and Bone mass (BM)] were extracted.

The maturity status of the young athletes was monitored using the anthropometric technique proposed by Moore et al. [28]. This technique uses chronological age and standing height to estimate age at maximum height velocity (APHV). The equation used was for males: APHV (years) = -7.999994 + (0.0036124 × (age × height)). This equation was used in males, whose categorization ranges in this sample were: -3APHV, -2APHV, -1APHV, 0APHV, +1APHV and + 2APHV.

The BSA (m^2^) was estimated by means of the DuBois equation, DuBois [23]. This equation uses weight and height and their corresponding allometric adjustments: BSA: = 0.007184*W^0.425^ * H^0.725^, where W = weight, H = standing height. Body Mass Index (BMI) was determined by the formula: BMI = weight (kg)/height^2^ (m).

### Statistics

The normal distribution of the data was verified by the Kolmogorov-Smirnov test. The study variables were analyzed using descriptive statistics of arithmetic mean (X), standard deviation (SD) and coefficient of variation (CV). Relationships between variables were obtained using Pearson’s correlation coefficient. Three simple and multiple stepwise regression models were developed to generate FFM prediction equations. The equations were analyzed using the following criteria: R^2^ (coefficient of determination), standard error of estimate (SEE), Collinearity (T-tolerance and variance inflation factor FIV for each independent variable). Models with an FIV < 10 or a tolerance greater than 0.1 were considered [29, 30]. The desirable reproducibility index (DRI) according to Lin’s [31] approach was also used. This approach evaluates the degree of agreement from the concordance correlation coefficient (CCC) in terms of precision (P) and accuracy (A). Using Lin’s approach [31], the concordance correlation coefficient (CCC) was calculated and interpreted as suggested by McBride [32] (near perfect > 0.99; substantial > 0.95–0.99; moderate = 0.90–0.95; and poor < 0.90). The models created were compared by means of the t-test for related samples. The significance level for all statistical tests was < 0.05. SPSS version 16.0 (IBM Corp., Armonk, NY, USA) and MedCalc 11.1.0 were used for statistical analysis.

## Results

Table 1 shows anthropometric and body composition variables aligned by decimal age and MS. The mean decimal age is 14.6 ± 1.7 years and the APHV (MS) was 14.12 ± 0.99 APHV. In both groups, it is observed that with increasing age and MS the anthropometric and body composition variables increase.

**Table 1:** Anthropometric and body composition characteristics of young basketball players aligned by chronologic age and APHV

Age (years)	Weight (kg)			Height (cm)		BMI (kg/m^2^)		BSA (m^2^)		BM (kg)		FM (kg)		FFM (kg)		%FM (%)	
	n	${\bf{\bar x}}$	SD	${\bf{\bar x}}$	SD	${\bf{\bar x}}$	SD	${\bf{\bar x}}$	SD	${\bf{\bar x}}$	SD	${\bf{\bar x}}$	SD	${\bf{\bar x}}$	SD	${\bf{\bar x}}$	SD
11	8	48,6	14,0	154,6	8,9	20,2	4,2	1,4	0,2	1,8	0,4	14,5	8,2	34,1	6,6	29,7	7,6
12	23	54,2	15,8	163,8	10,6	19,9	3,5	1,6	0,3	2,0	0,5	14,7	7,6	39,6	9,1	27,0	7,1
13	26	59,2	11,1	170,4	9,4	20,3	2,3	1,7	0,2	2,4	0,4	12,5	4,5	47,2	9,3	21,8	6,2
14	24	66,4	12,5	178,7	9,2	20,6	2,3	1,8	0,2	2,9	0,5	11,5	3,6	54,9	9,3	17,9	3,3
15	24	77,7	10,7	185,3	9,5	22,6	2,5	2,0	0,2	3,4	0,4	13,7	4,4	63,6	7,6	18,2	3,7
Total	105	63,2	15,6	173,1	13,3	20,8	3,0	1,8	0,3	2,6	0,7	13,2	5,5	50,0	12,9	21,8	6,8
MS (APHV)																	
-3	4	41,2	0,9	146,9	6,8	19,2	1,6	1,3	0,1	1,5	0,2	12,5	1,4	28,7	2,1	31,6	3,8
-2	10	44,1	4,4	155,6	4,3	18,2	2,0	1,4	0,1	1,7	0,2	10,6	2,5	33,5	2,8	24,8	4,4
-1	16	52,2	10,4	160,7	6,2	20,1	3,2	1,5	0,2	1,9	0,3	15,3	6,3	36,9	5,6	29,6	6,8
0	53	64,3	11,9	175,4	7,6	20,8	2,8	1,8	0,2	2,7	0,4	12,5	6,0	52,1	7,9	19,6	5,8
1	16	79,5	11,3	185,0	7,2	23,2	2,6	2,0	0,2	3,4	0,3	14,7	4,9	64,9	7,3	19,0	4,1
2	6	83,7	8,5	197,1	4,9	21,6	2,3	2,2	0,1	3,5	0,4	13,9	3,3	67,0	8,6	17,8	2,8
Total	105	63,5	15,6	173,3	13,3	20,8	3,0	1,8	0,3	2,6	0,7	13,2	5,5	50,3	12,9	21,8	6,8

Legend: X: mean, SD: standard deviation, BMI: body mass index, BSA: body surface area, APHV: peak growth velocity years, BM: bone mass, FM: fat mass, FFM: fat-free mass and percentage of fat mass (%FM), MS: maturity stage, APHV: age at maximum height velocity (APHV)

The simple linear regression values are shown in Table 2. All five variables (age, APHV, weight and BSA) evidenced positive relationships with FFM (DXA). These ranged from 0.74 to 0.96 (*p* < 0.001). The highest explanatory power was observed with BSA (R^2^ = 92%), followed by body weight (R^2^ = 87%). In addition, it was observed that APHV showed a better predictive percentage in relation to decimal age (R^2^ = 76%).

**Table 2:** Multiple linear regression values predicting FFM in young basketball players

Dependent variable	Independent variable	R	R^2^	SEE	*p*
FFM (DXA)	Age (years)	0.74	0.55	8,73	0.0001
	MS (APHV)	0.85	0.76	6,87	0.0001
	Weight (kg)	0.93	0.87	4,76	0.0001
	BSA (m^2^)	0.96	0.92	3,78	0.0001

Legend: MS: maturity stage, BSA: body surface area, APHV: age at peak height velocity, SEE: standard error of estimation

Table 3 shows the equations proposed to estimate the FFM. In general, 3 regression equations were developed. The first uses age and body weight, the second uses age and BSA and the third uses APHV and BSA. Tolerance values of 0.34 to 0.66 and FVI values of 1.52 to 2.98 were evident in all 3 equations. No collinearity was observed in the 3 equations. However, the explanatory power of equations 2 and 3 (R^2^ = 94%) were superior in relation to equation 1 (R^2^ = 92%), respectively.

**Table 3:** Development of regression equations estimating the FFM in young basketball players

N	Equations	Collinearity		R	R^2^	SEE	*p*
		T	FIV				
1	-30.059+(2.926*age)+(0.625*Weight)	--	--	0.96	0.92	3.59	0.0001
	Age	0.66	1.52				
	Weight	0.66	1.52				
2	-45.719+(1.934*age)+(39.388*BSA)	--	--	0.97	0.94	3.27	0.0001
	Age	0.57	1.76				
	BSA	0.57	1.76				
3	-15.284+(1.765*APHV)+(37.610*(BSA)	--	--	0.97	0.94	3.50	0.0001
	APHV	0.34	2.98				
	BSA	0.34	2.98				

Legend: BSA: Body surface area, APHV: age at peak height velocity, T: Tolerance, SES: Standard errors of estimation

Table 4 shows the comparisons between the reference DXA with the three equations and the DRI values of the proposed equations. There were no significant differences between equations 1, 2 and 3 with the reference method (*p* > 0.001). However, equations 2 evidences a lower CV (4.98%) in relation to the other equations. In general, the three equations present similar CCC (0.96), precision (0.96) and accuracy (0.99), respectively.

**Table 4:** Mean values, SD and CCC describing the agreement between the reference method (DXA) and the equations generated

Equations	${\bf{\bar x}}$	SD	t-test	*p*		DRI		
					CV	CCC	*P*	A
DXA Reference (FFM)	50.0422	12.92	--	--	--	--	--	--
Equation 1	50.0404	12.42	-0.005	0.995	5.53	0.96	0.961	0.999
Equation 2	50.9086	12.64	2.74	0.007	4.985	0.96	0.968	0.997
Equation 3	50.1902	12.42	-0.002	0.998	5.018	0.962	0.963	0.999

Legend: ${\bf{\bar x}}$: mean, SD: standard deviation, FFM: fat-free mass, DRI: desirable reproducibility index, CV: coefficient of variation, CCC: concordance correlation coefficient, P: precision, A: accuracy

## Discussion

The aim of the study was to validate the BSA as an anthropometric indicator to estimate the FFM of young basketball players. Age, body weight, MS or BSA were used as predictor variables. In fact, the results have shown that the best predictor of FFM was BSA. From these results, three regression equations estimating FFM in young basketball players were generated.

The three equations developed showed high explanatory power. For example, equation 1 using age and weight explained 92%. However, equation 2 (using age and BSA) and equation 3 (using APHV and BSA) as predictors, explained 94%. In addition, in the three cases we did not observe collinearity, since tolerance and FIV presented values within the established limits [33].

When comparing the reference DXA method with each of the equations, no significant differences were found. However, equation 2, that uses age and BSA, is the one that presents a lower SEE and CV than equations 1 and 3, respectively. Therefore, to ratify this pattern observed in the equations, we used the Desirable Reproducibility Index (DRI) proposed by Lin [31], where the CCC, precision and accuracy were similar in the three equations. In fact, these findings are relatively similar with other studies that sought similar targets in schoolchildren [34] and young athletes [18, 35, 36]. However, the results obtained here presented a higher R^2^ and high values of precision and accuracy.

Consequently, these results obtained in this study confirm that age and BSA are excellent predictors of FFM, so they should be included in the process of body composition assessment of young basketball players. Although the use of equation 3 using the MS (APHV) and BSA are not ruled out since the explanatory power is relatively similar to equations 1 and 2, respectively. In fact, BSA has been widely accepted as the most appropriate biometric unit to normalize physiological indices related to body metabolism in individuals of different body sizes [37]. Well, a recent study has evidenced a high positive relationship between MS determined by anthropometry with BSA in children and adolescents and its possible use as a predictor of MS [38]. Because BSA is an indicator of metabolic mass that is less affected by abnormal adipose tissue [39] due to the scaling of both weight and height.

Another recent study has verified the usefulness of the BSA to predict FFM in young 3 × 3 basketball players (determined by bio-impedance), since they have verified a better association in relation to body mass index and tri-ponderal index [40], so its use and application in young basketball players is determinant.

The estimation of FFM through BSA in young basketball players in this study can help in the evaluation, monitoring and in the design of training programs, so FFM basically intervenes in the production of force, especially during jumps, sprint and changes of rhythm [41].

Overall, FFM can provide useful information for sports scientists and coaches to improve the body composition of athletes [42]. As well as guide coaching staff decisions and assist with short-, medium-, and long-term nutritional goals.

It is widely known that basketball is characterized by evidencing high physical and physiological demands [43]. Therefore, it is necessary to ensure a favorable body composition profile (e.g., less fat mass and higher FFM), which would greatly benefit athletes in their physical performance [44].

Fat-free mass is the most important factor in the body composition of athletes, as it is involved in force production. This intervenes in many aspects of basketball, for example, in sprinting, defense and jumping [45]. This provides the basis for sport-specific technical skills and locomotor activities [46].

Therefore, players with higher FFM can jump higher than their counterparts who have higher fat mass [47, 48]. Which contributes to largely to optimize physical performance, motor ability of basketball players, prevent injuries and improve the health of athletes, especially during the stage of physical growth and biological development.

In general, this study presents some strengths that should be highlighted, for example, it is one of the first studies that considers BSA as a predictor of FFM in young basketball players. For BSA is characterized as a better indicator of metabolic mass than body weight [49]. This is often used to determine basal metabolic rate and recommended caloric intake for individuals at different stages of life and with different levels of physical activity [50]. Furthermore, the validation of equation 2 is assured, since we used a standardized protocol (standard other) such as DXA.

It must also be recognized that the study has some weaknesses. These are related to the cross-sectional design used since it does not allow cause and effect relationships. In addition, it was not possible to evaluate physical tests related to muscle strength, and it is also necessary to explore other BSA formulas. It is suggested that future studies consider these variables to obtain promising results in young basketball players.

## Conclusion

In sum, this study concludes that body BSA is a better predictor of FFM in relation to body weight and MS in young basketball players. The results suggest the use of the anthropometric equation using decimal age and BSA to estimate FFM in young basketball players. This new validated method can be used to design, evaluate, and control training programs, and monitor the weight status of athletes. Its calculations can be performed at the following link: http://reidebihu.net/ffmbasketbrasil.php.

## Data Availability

The study data may be requested from the corresponding author, who will make the database available.

## Abbreviations

APHV: Age at peak height velocity
BM: Bone mass
BMI: Body mass index
BSA: Body Surface Area (BSA)
DXA: Dual X-ray absorptiometry
FFM: Fat Free Mass
FM: Fat mass
MS: Maturity status
